# *Chimonanthus salicifolius* extract alleviates DSS‐induced colitis and regulates gut microbiota in mice

**DOI:** 10.1002/fsn3.3282

**Published:** 2023-03-16

**Authors:** Lin Chen, Xin Li, Qing Gu

**Affiliations:** ^1^ Key Laboratory for Food Microbial Technology of Zhejiang Province, Zhejiang Gongshang University Zhejiang Business College Hangzhou China; ^2^ Research and develop department Zhejiang Tact Artiste Biotechnology Group Co. Ltd Hangzhou China

**Keywords:** *Chimonanthus salicifolius*, colitis, gut microbiota, *Lactobacillus*

## Abstract

Ulcerative colitis is a chronic and recurrent gastrointestinal intestinal disease accompanied by inflammatory disorders, immunologic inadequacy, and intestinal flora dysbiosis, and current therapeutic pharmaceuticals have limited side effects. In this study, we revealed the extraction method of *Chimonanthus salicifolius*, analyzed the main component, compared the effect of its extract, *Lactobacillus*, and conventional drugs with different properties on DSS (dextran sodium sulfate)‐induced colitis, and indicated extract regulatory properties of inestinal flora. A colitis model was established on experimental design, and BALB/c mice (male, 7 weeks old) were randomly assigned to five groups (*n* = 10): control, DSS model, *Chimonanthus salicifolius* extract (CSE), *Lactobacillus rhamnosus* GG (LGG), and 5‐aminosalicylic acid (5‐ASA) groups. The three treatments could alleviate the symptoms and remit inflammation induced by DSS, in which CSE and LGG groups could both decrease the proinflammatory cytokine IL‐6, IL‐8, and TNF‐α levels and increase anti‐inflammatory cytokines IL‐10 and TGF‐β. The CSE intervention significantly promoted the higher production of butyric acid than LGG and 5‐ASA groups (*p* < .05) after DSS challenge. Analysis of intestinal flora showed that CSE administration remarkably decreased the relative abundance of pathogenic bacteria *Heliobacteriaceae* and *Peptococcaceae* and exhibited higher abundance of *Lactobacillaceae* and *Bifidobacterium* than LGG in intestinal tract of mice (*p* < .05). These findings indicated that *Chimonanthus salicifolius* extract may have been beneficial for preventing and treating colitis.

## Graphical abstract

The intervention of *C. salicifolius* extract on colitis mice effectively regulated intestinal flora induced by DSS and restored their relative abundance. The extract group significantly increased the abundance and diversity of Lactobacillaceae compared with other treatments. Therefore, the results indicate that *C. salicifolius* extract presents the potential to be developed as a candidate for treating colitis.

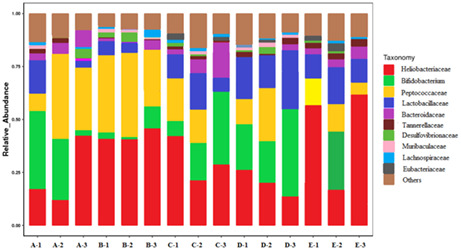

## 1 INTRODUCTION

Inflammatory bowel disease (IBD) is a colonic inflammatory disease which causes dyspepsia, nutritional obstacle, disgusting vomit,even can induce lower abdomen colic and hematochezia symptoms (Cho, 2008; Kaser, 2009; Zhong et al., 2021). According to different etiology, IBD is divided into ulcerative, ischemic, and pseudomembranous colitis, of which ulcerative colonic (UC) accounted for the highest proportion (Buono et al., 2017; Chey et al., 2016; Lacy & Moreau, 2016). In recent years, epidemiological reports indicated an increasing incidence of IBD, with about 10%–20% of the population has affected quality of life caused by colitis, which has become one of the most common diseases in the world (Gwee et al., 2017; Krogsg et al., 2013). Routine medications for IBD include antibiotic and aminosalicylic acids, glucocorticoids, and immunosuppressants, in which 5‐ASA is the main treatment for maintenance and remission of IBD (Aljammaz et al., 2020; Sugaya et al., 2021). However, 5‐ASA derivatives are easily absorbed by the duodenum and upper jejunum and cannot reach the lesion site which lost its topical anti‐inflammatory in the intestinal mucosa, meanwhile, the vast majority of patients present with alternating recurrence and remission and adverse reactions to drugs may cause headache, vomiting, and nausea (Arokiadoss & Weber, 2021; Fatahi et al., 2022). Probiotic was reported to have therapeutic properties, such as improving microbiota and regulating immune response. Administration of *Lactobacillus rhamnosus* GG, significantly relieved DSS‐induced colitis, its effect was related to altering the expression of the MUC and TFF genes. The soluble‐protein HM0539 derived from LGG, showed significant protective effects against murine colitis, the protective function involved in the modulation of inflammatory responses (Li et al., 2020; Macdonald et al., 2011). Therefore, exploring safe therapeutic targets with higher efficacy and lower incidence of adverse reactions is still a focus of attention in the treatment of IBD.

Although the etiology and etiopathogenesis of UC are not completely elucidated, intestinal mucosal barrier impairment is considered to be an important pathogenesis of IBD; with the long‐term intestinal inflammation, the disruption of intestinal mucosal barrier function can lead to the displacement of intestinal bacteria and endotoxins and the release of inflammatory mediators, thus resulting in persistent tissue damage (Ozair et al., 2021; Staudacher et al., 2021; Wójcik et al., 2020). It is becoming increasingly clear that a combination of susceptibility genes, environmental factors, and the immune system plays important roles in production of inflammatory cytokines, and mediators cause inflammation of the intestinal mucosa, in which intestinal tract environmental factors are primary (Kumar et al., 2021; Luiza et al., 2020; Tilg & Kaser, 2011). The gut microbiome constitutes a vast and complex ecosystem in intestinal tract environment, in which the bacteria are mainly composed of *Bacteroidetes*, *Firmicutes*, *Proteobacteria*, and *Actinobacteria* (Agnello et al., 2020; Labus et al., 2017). Most of these bacteria belong to obligate anaerobe; *Firmicutes* and *Bacteroidetes* accounted for more than 90% of intestinal bacteria (Belizário & Faintuch, 2018). The species and quantity of intestinal flora vary with intestine location. When the structure and quantity of intestinal flora change, it can affect the homeostasis of the intestinal environment and the abnormal function of intestinal flora, which then leads to intestinal tract diseases (Lin et al., 2021; Zhou et al., 2020). According to previous research, in colitis patients, compared with healthy people, the number of microorganisms in the intestinal tract is severely reduced, especially for the benefit of bacteria (Xing et al., 2020). With the decreasing abundance and diversity of the bacteria, the stability of the dominant bacteria is damaged in the intestinal tract of patients. The imbalance of intestinal flora has a certain relationship with the incidence and aggravation of colitis (Tang et al., 2021).

*Chimonanthus salicifolius* (*C. salicifolius*) is a unique medicinal plant that belongs to the traditional antiviral of She nationality in China, which is classified as *Calycanthaceae* family and applied for food (Liang et al., 2016; Liu & Fu, 2018). According to the Chinese Pharmacopeia, it is a kind of food herbal tea used to treat stomach pain and indigestion caused by dyspepsia and eating injury, as well as to prevent infantile malnutrition and diarrhea (Chen, Jiang, et al., 2017; Chen, Ouyang, et al., 2017). The genome of *C. salicifolius* was sequenced as 820.1 Mb containing 36,651 annotated protein‐coding genes (Lv et al., 2020). The chemical constituents mainly focused on volatile oil (monoterpenoids and sesquiterpenoids), and its stems and leaves contained coumarins, flavonoids, anthraquinones, and other components, which have antioxidant and anti‐inflammatory function (Wang et al., 2016). The current studies mainly researched its chemical constituents, but are rarely reported on its bacteriostasis application, especially for gastrointestinal regulation. In our previous study, *C. salicifolius* reduced defecation time and significantly increased gastrointestinal transit rate, fecal particles, and water content, which effectively improved loperamide‐induced constipation in mice. The aim of this study was to identify the main components of *C. salicifolius* extract, verify the extract treatment effects on DSS‐induced colitis comparison with *Lactobacillus* and 5‐ASA evidenced by SCFA and cytokine levels, and reveal the regulatory mechanisms of intestinal microbiome.

## 2 MATERIALS AND METHODS

### 2.1 Materials and animals

*Chimonanthus salicifolius* leaves were obtained from Zhejiang TactArtiste Biotechnology Group Co. Ltd, 5‐amino salicylic acid (5‐ASA) from Shanghai ethypharm co., LTD Company Limited (Batch No. 160209), and dextran sodium sulfate (DSS relative molecular mass: 36,000–50,000 Mw) from MP Biomedicals, America. ELISA Kit was obtained from Shenzhen Dakewei Biotechnology Co., LTD. The standard substance was purchased from Sigma‐Aldrich, USA, such as acetic acid, propionic acid, isobutyric acid, butyric acid, and valeric acid. *Lactobacillus rhamnosus* GG (LGG) was isolated from commercial fermented milk products (Yili, Inner Mongolia).

The animal experiments were conducted in strict according to the European Community guidelines. The protocol was reviewed and approved by the Ethics Committee of Zhejiang Chinese Medical University, China (JN. No 202110716).

### 2.2 Preparation of *C. salicifolius* aqueous extract (CSE)

*Chimonanthus salicifolius* leaves were obtained from Zhejiang TactArtiste Biotechnology Group Co. Ltd. The fresh leaves, which were prepared for crushing in burnisher, were dehydrated at room temperature with constant aeration. Three liter of sterile water was added into 500 g dry material and then boiled at 95°C for 3 h in a water bath. The supernate was collected and filtered by kieselguhr. The extract was centrifuged (8,000 *g* for 15 min), supernatant was collected, and freeze‐dried by freeze dryer (Christ Delta LSC). Then, the extracted water was redissolved with aquae sterilisata into 0.5 g/mL of crude drug. Mice were administrated intragastrically by gavage needle with *C. salicifolius* extract twice a day by 10 mL/kg bodyweight (equivalent to 10 g/kg).

### 2.3 High‐performance liquid chromatography (HPLC) analysis

Main component of *C. salicifolius* extract was determined by high‐performance liquid chromatography (HPLC) equipment. CSE (0.1 g/mL) was prepared in a 10 mL volumetric flask, where methanol was added to dissolve and dilute the solution to the scale. After mixing the solution, the sample was filtered by 0.45 μm microporous membrane (Sigma). A ZORBAX Extend‐C18 liquid chromatography column (250 mm × 4.6 mm, 5 μm) was used for separation and the temperature was maintained at 25°C. Ten microliter of the sample was injected into the system with a 1 mL/min flow rate. The mobile phase consisted of acetonitrile (A) and 0.2% glacial acetic acid water (B). The linear gradient elution started at 14.5%–18.0% (A), changed to 16.5%–35% (A) after 20 min, and changed to 50%–16.5% (A) after 30 min. The extract was monitored by a diode array detector at 365 nm.

### 2.4 Induction of colitis and experimental design

A total of 50 male BALB/c mice (7‐week‐old, bodyweight 30–32 g), provided by the Shanghai Laboratory Animal Center, were housed in a standard rodent cage at a constant temperature and humidity under conventional standard laboratory conditions. All protocols for this study were approved by the Laboratory Animal Ethics of Zhejiang Chinese Medical University, China. *Lactobacillus rhamnosus* GG was cultured and propagated twice in MRS broth at 37°C for 16 h, and then the bacterial suspension was collected and adjusted to 3.0 × 10^9^ CFU/mL with normal saline which would be used for oral gavage to mice.

The mice (BALB/c mice, male, 7 weeks old, inbred strain) were randomly divided into five groups (*n* = 10 for each group) and classified into a normal control group (control), DSS‐induced model group (2.5%, w/v, 36–50 k Da), 10 g/kg CSE group, LGG (3.0 × 10^9^ CFU/mL), and 5‐ASA (100 mg/kg) group (Murray et al., 2020; Wen et al., 2021). Apart from the control group, DSS solution (2.5%, w/v) was prepared for drinking water to the mice daily in the other four groups for 2 weeks, while the mice in the control group were given a saline solution. After administering, in the model groups were detected changes in colon length, occult blood, and hematoxylin–eosin (HE) staining sections. When the model groups were conducted, the treatment with CSE, LGG, and 5‐ASA was intragastrically administered daily to the mice for 14 days, meanwhile, the control and DSS groups were given 0.85% normal saline. At the end of the experiment, animals were anesthetized to death and the colon of each mouse was collected. The upper part of the colon below 1 cm was taken 2 cm and cut into five sections for inflammatory cytokines, short‐chain fatty acid, and microbiome assay.

### 2.5 Histological analysis of colon

The distal colon was fixed at 1 cm in 10% formalin for 16–18 h, then rinsed with water for 24 h to remove the fixative fluid. Different gradient ethanol was used as a dehydrating agent to dehydrate colon tissue. The dehydrated colon tissue was placed in an equal mixture of alcohol and xylene for 15 min by EG1160 paraffin‐embedding machine (Leica) for paraffin embedding. The standard for evaluation of histological injury was identified by HE‐staining sections.

### 2.6 Determination of cytokine levels in colon tissue by real‐time PCR

The sample isolated from 30 mg colon tissue was used for extracting RNA using the TRIzol™ reagent. The total RNA of mouse tissue was reversely transcribed into cDNA using the PrimeScript™ First Strand cDNA Synthesis Kit (Thermo Scientific). The reverse transcription system was as follows (10 μL): 6 × buffer 2 μL, prime mix 1 μL, enzyme 1 μL, and RNA + RNase Free dH2O 6 μL. Specific primers of cytokine are shown in Table 1. Real‐time fluorescence quantitative PCR (RT‐QPCR) system was prepared according to the requirements, and the BIO‐RAD real‐time system (Bio‐Rad Laboratories) was used for RT‐QPCR. The expression level of the target gene was calculated using the 2^−ΔΔCt^ method (Johnson et al., 2005; Schmittgen & Livak, 2008).

**TABLE 1:** Primers used in real‐time PCR.

Target gene	Sequence (5′‐3′)	Sequence (3′‐5′)
IL‐6	CCAAGAGGTGAGTGCTTCCC	CTGTTGTTCAGACTCTCTCCCT
IL‐8	CGGCAATGAAGCTTCTGTAT	CCTTGAAACTCTTTGCCTCA
TNF‐α	CTGAACTTCGGGGTGATCGG	GGCTTGTCACTCGAATTTTGAGA
IL‐10	GACAATACTGCTAACCGACT	ATCACTCTTCACCTGCTCCAC
TGF‐β	CCACCTGCAAGACCATCGAC	CTGGCGAGCCTTAGTTTGGAC

### 2.7 Determination of short‐chain fatty acid (SCFA)

The tissue sample was precisely weighed 50 mg, added into 1 mL of 6% phosphoric acid solution homogenate, and then transferred to a 20 mL headspace sample bottle. GC–MS 7000D (Agilent Technologies) was used to measure the changes of short‐chain fatty acids in the colon of mice for each intervention group. Static headspace injection was used with an incubation temperature of 85°C, incubation time of 30 min, injection needle temperature of 95°C, and injection volume of 1 mL. The selective ion monitoring mode was adopted. The mass‐to‐charge ratio of acetic acid, propionic acid, isobutyric acid, butyric acid, and valeric acid were 60, 74, 743, 60, 60 respectively. The collected peak area ratio of each peak was substituted into the standard curve to calculate the content of each component in the sample.

### 2.8 Analysis of intestinal microflora

The colon sample was taken from the fresh colon tissue treated with liquid nitrogen, which ground with a sterile mortar. Total DNA in colon samples was extracted using the Power Soil DNA Isolation Kit (MO BIO, Cat. No. 12888). DNA purity and concentration were detected by agarose gel electrophoresis. After quantifying DNA, a polymerase chain reaction (PCR) analysis of the V4 variable region of the bacterial 16 S rRNA was amplified used specific primer: 515F(5′‐GTGCCAGCMGCCGCGGTAA‐3′) and 806R(5′‐GGACTACHVGGGTWTCTAAT‐3′). The PCR product was confirmed by using 1% agarose gel electrophoresis. The amplified products were purified with Beckman DNA Clean Beads and quantified by the Qubit 2.0 fluorimeter (Invitrogen; Qiu et al., 2018). Lastly, the PCR purified products were sequenced on an Illumina HiSeq 2500 platform (Illumina) according to the protocol described. Sequences with 97% similarity cutoff were assigned to the same OTUs. OTUs abundance information was normalized using a standard of sequence number corresponding to the sample with the least sequences. Principal coordinate analysis (PCoA) was used to analyze the diversity between groups. The alpha diversity was calculated to analyze the diversity of the microbial community by using QIIME (Version 1.7.0). R software (Version 2.15.3) was used to determine the beta diversity and compare the differences between treatment groups (Bokulich & Spiller, 2016).

### 2.9 Statistical analyses

Statistical analysis of the data was calculated and conducted with GraphPad 9.3 Software. Significant differences among groups were performed using one‐way ANOVA. The data were expressed as the mean value ± SD. *p* < .05 was regarded as statistically significant.

## 3 RESULTS AND DISCUSSION

### 3.1 Main components of CSE

**FIGURE 1:**
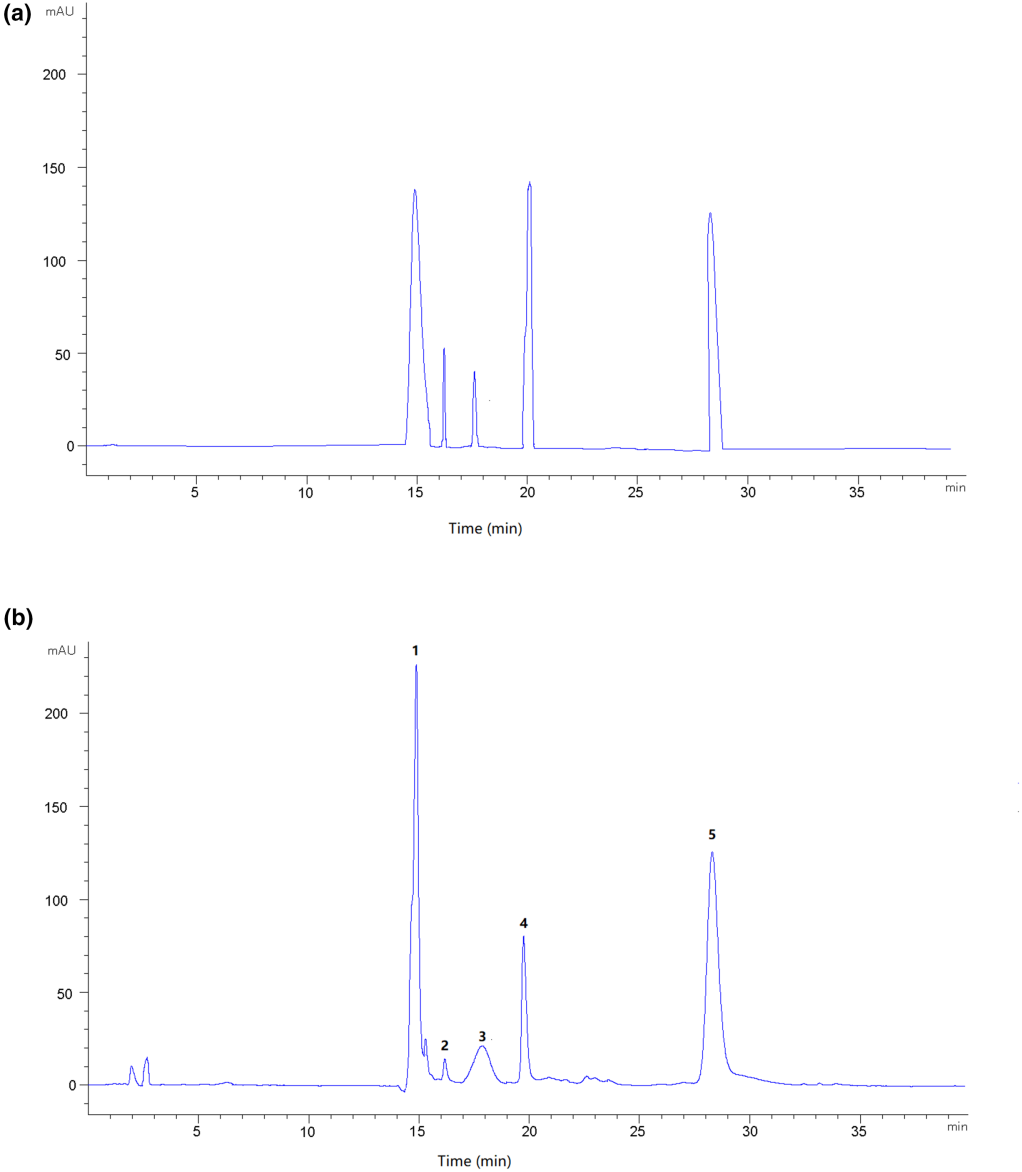
HPLC analysis of CES (a) and mixed standard samples (b). The peak identifications are (1) rutin; (2) hyperin; (3) isoquercitrin; (4) afzelin; and (5) kaempferol.

The HPLC results was represented in Figure 1. The main components of CSE were rutin, hyperin, isoquercitrin, afzelin, and kaempferol. The instrument precision inspection showed that the standard deviations were 0.56%, 0.23%, 0.62%, and 0.56%, respectively. The data were less than 2%, which indicates the precision of the instrument. The content of rutin, hyperin, isoquercitrin, afzelin, and kaempferol in CSE were 56.34, 12.76, 26.58, and 13.45 mg/g, respectively.

### 3.2 *Chimonanthus salicifolius* extract improved the colonic damage induced by DSS

DSS‐induced colitis model mice were measured by bodyweight of mice, the fecal occult blood, and gross blood stool. Changes in mice weight are observed in Table 2.

**TABLE 2:** Weight change of DSS‐induced colitis in mice (Mean ± SD, *n* = 10).

	Day 1	Day 7	Day14	Day 21	Day 28
Control	31.35 ± 1.28	32.96 ± 2.23	34.88 ± 2.05	36.23 ± 1.15	38.56 ± 1.23
DSS	31.40 ± 1.25	32.78 ± 1.75	28.56 ± 1.50**	27.85 ± 2.16**	27.98 ± 1.06**
DSS/LGG	31.38 ± 1.56	32.79 ± 2.14	28.53 ± 1.78**	28.88 ± 1.15**	29.02 ± 3.25**
DSS/CES	31.36 ± 0.88	32.78 ± 1.22	28.56 ± 0.97**	28.92 ± 1.13**	29.12 ± 3.25**
DSS/5‐ASA	31.34 ± 2.36	32.90 ± 3.56	28.50 ± 1.06**	28.65 ± 1.48**	29.00 ± 1.25**

*Note*: Data show the mean ± SD (*n* = 10), and mean values with ** are significantly different (*p* < .05).

During the first 7 days, there was no significant difference in bodyweight between the treatment groups. On day 14, mice weight of DSS treatments reduced significantly, meanwhile accompanied with mice's curly hair and lazy phenomenon. The stool of the mice with weight loss was irregular and adhered to the anus; stool test was positive with fecal occult blood test paper, which showed that the DSS‐induced colitis model was successfully constructed. After gavage by 10 g/kg CSE, LGG (3.0 × 10^9^ CFU/mL), and 5‐ASA (100 mg/kg), respectively, for 14 days, significantly reduced weight after DSS treatment compared with control group. There were no significant differences in bodyweight between the DSS treatment groups.

**FIGURE 2:**
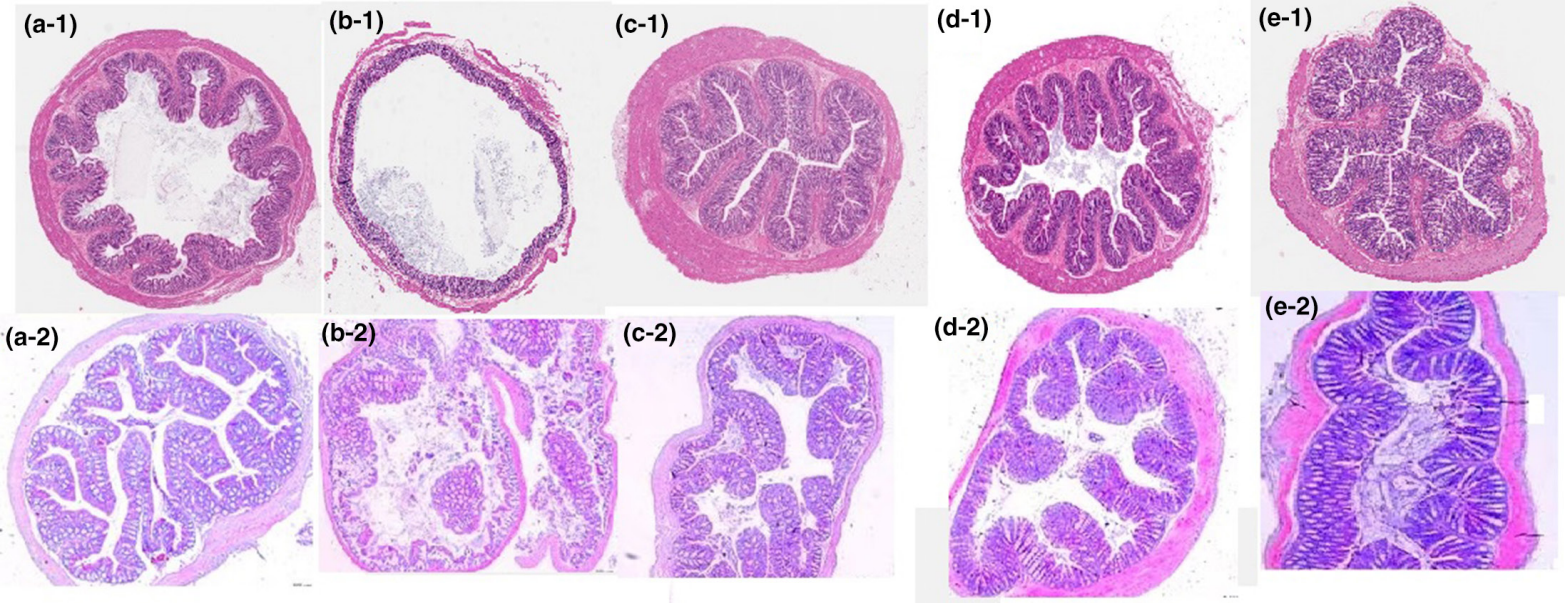
Histological scores of treatments. (a): Control; (b): model; (c): LGG; (d): CSE; and (e): 5‐ASA;

Histopathological observation was an effective method to diagnose the severity of colitis. In order to evaluate the protective function of different treatments, the histological scores of colonic tissues were based on inflammatory cells, goblet cells, crypt arrangement, epithelial villous, and glandular structure in mice colon. The histological score was defined as the sum of score for five indexes (Table 3). In the control group (Figure 2), the colon epithelium, villi structure, and crypts were arranged orderly, and there were abundant goblet cells. The glands were intact, and no inflammatory cell infiltration was observed. However, histological score of the DSS model was 11.78 ± 1.75, ulceration appeared in the mid‐intestinal tissue, intestinal glandular, epithelial structures, and villi and crypts completely disappeared with extensive connective tissue proliferation in the mucosal layer. In the CSE group (histological scores 9.13 ± 1.06), a spot of connective tissue proliferation appeared, but villi, crypts, and intestinal glandular were relatively complete, which showed a significant decreased scores from LGG and 5‐ASA groups. The intestinal glandular of LGG group was disorderly and inflammatory cell emerged in 5‐ASA group.

**TABLE 3:** Effects of different treatments on the colonic damage induced by DSS.

Treatment	Control	DSS	DSS/LGG	DSS/CES	DSS/5‐ASA
Colon length	7.68 ± 0.28^a^	5.24 ± 0.23^c^	5.96 ± 0.76^c^	6.12 ± 1.15^bc^	5.86 ± 0.76^c^
Histological score	0.60 ± 1.00^a^	11.78 ± 1.75^c^	10.56 ± 0.90^c^	9.13 ± 1.06^bc^	10.98 ± 1.06^c^

*Note*: The data are in accordance with normal distribution. Data are shown as mean ± SD (*n* = 10). Different letters in each row indicate a significant difference (*p* < .05) among groups.

Colon length could reflect DSS‐induced colonic damage. After DSS exposure, colon lengths of treatments were shorter than control group, but the colon length of mice supplemented with CSE was a significant increase compared to LGG and ASA groups, which indicated that *C. salicifolius* extract could alleviate DSS‐induced colitis.

### 3.3 Expression levels of cytokine genes in colon tissue

Effects of different treatments on the relative expression of cytokines in colonic tissue were analyzed by real‐time PCR. IL‐6 and IL‐8 can stimulate the proliferation of T and B cells and secrete antibodies to participate in the inflammatory response. TNF‐α is a recognized proinflammatory cytokine, which activates and induces the production of inflammatory cells. According to previous studies, these cytokines are commonly used as proinflammatory factors to evaluate immunomodulatory function (Kobayashi et al., 2017; Liu et al., 2020).

We detected levels of changes in proinflammatory cytokines (IL‐6, IL‐8, and TNF‐α) and anti‐inflammatory cytokines (IL‐10 and TGF‐β) in colon tissues of mice (Table 4). Administration of the different treatments could drastically decrease inflammatory cytokines expression (IL‐6, IL‐8, and TNF‐α) and increase anti‐inflammatory (IL‐10 and TGF‐β) compared to model group. There was no significant difference in TNF‐α levels between the control and treatment groups. Notably, the LGG group showed the lowest expression level of IL‐6, and administration of the CSE group could significantly reduce IL‐8 expression level comparison with LGG and 5‐ASA groups. The CSE group increased the expression of anti‐inflammatory cytokines significantly in comparison with other two groups and presented the highest expression at IL‐10 and TGF‐β. Overall, the administration of CSE suggested the most significant inflammatory modulation effect.

**TABLE 4:** Effects of different treatments on the relative expression of cytokines in colonic tissue.

Cytokines	Control	DSS	DSS/LGG	DSS/CES	DSS/5‐ASA
IL‐6	1.21 ± 0.39^a^	60.56 ± 11.42^c^	8.54 ± 2.76^ab^	29.65 ± 2.87^bc^	30.62 ± 5.68^c^
IL‐8	1.23 ± 0.17^a^	10.21 ± 1.25^c^	4.89 ± 2.76^b^	3.08 ± 0.67^ab^	4.86 ± 0.12^b^
TNF‐α	3.38 ± 0.85^a^	9.12 ± 5.21^b^	4.42 ± 0.56^a^	4.86 ± 0.56^a^	4.67 ± 0.23^a^
IL‐10	1.25 ± 0.68^a^	0.56 ± 0.58^b^	2.76 ± 1.76^ab^	6.37 ± 1.56^b^	1.02 ± 0.78^a^
TGF‐β	1.85 ± 0.65^a^	0.35 ± 0.17^c^	0.48 ± 0.16^c^	0. 65 ± 0.16^bc^	0.38 ± 0.12^c^

*Note*: Data are shown as mean ± SD (*n* = 10). Different letters in each row indicate a significant difference (*p* < .05) among groups.

### 3.4 *Chimonanthus salicifolius* extract regulated the production of SCFAs

Short‐chain fatty acids (SCFAs) degraded undigested carbohydrates and small amounts of protein, which are produced primarily by microorganisms that are essential for gut health. In the model group, the level of acetic acid (AA), propionic acid (PA), isobutyric acid (IA), butyric acid (BA), and valeric acid were (VA) significantly lower than control group (Table 5). For AA and PA index, 5‐ASA group showed no significant difference compared with the model group. The LGG group presented the highest value of AA (3.02 ± 0.16) and PA (152.87 ± 10.24). However, the value of IA was similar among the three treatments. The CSE group produced the highest value (176.56 ± 21.78) in BA. The VA value of 5‐ASA group significantly increased compared with the other treatments. These results showed that LGG and CSE treatment could recover the decrease in SCFAs caused by DSS induced colitis.

**TABLE 5:** Effects of different treatments on SCFAs in colonic tissue.

SCFA	Control	DSS	DSS/LGG	DSS/CES	DSS/5‐ASA
Acetic acid	1.85 ± 0.13^a^	0.89 ± 0.23^b^	3.02 ± 0.16^c^	2.98 ± 0.54 ^c^	1.02 ± 0.26^b^
Propionic acid	390 ± 30.23 ^a^	125.34 ± 11.46 ^c^	152.87 ± 10.24 ^b^	145.87 ± 13.54 ^b^	328.78 ± 20.07^a^
Isobutyric acid	15.02 ± 0.43^a^	7.35 ± 0.76^b^	9.89 ± 0.89^c^	9.98 ± 0.65^c^	9.93 ± 0.67^c^
Butyric acid	224.25 ± 12.04^a^	85.45 ± 21.45^d^	165.46 ± 15.56^b^	176.56 ± 21.78^c^	170.46 ± 12.65^c^
Valerate acid	8.52 ± 0.14^a^	12.23 ± 1.89^b^	14.48 ± 0.16^c^	14. 65 ± 0.15^c^	18.23 ± 0.97^d^

*Note*: Data are shown as mean ± SD (*n* = 10). Different letters in each row indicate a significant difference (*p* < .05) among groups.

### 3.5 Effect of *C. salicifolius* extract on the alpha and beta diversities of colon

**FIGURE 3:**
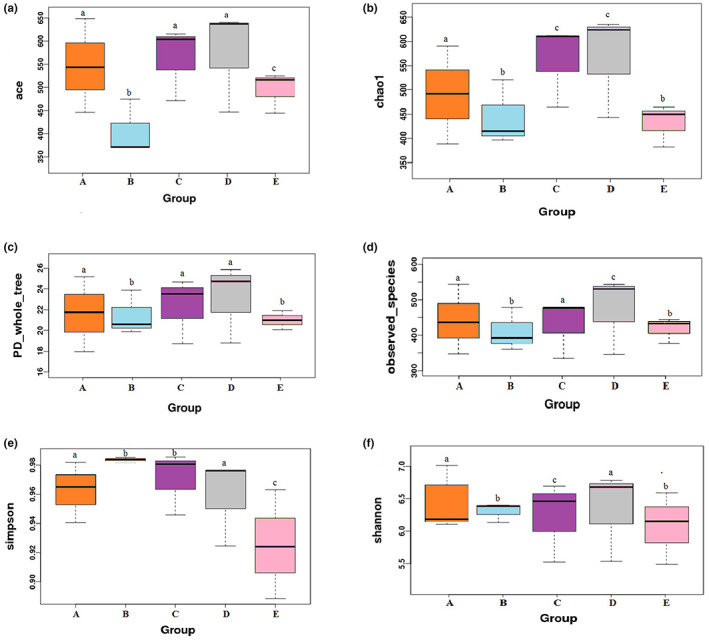
Alpha diversity indexes analysis of microbial communities. (a): ACE; (b): Chao1; (c): PD whole‐tree; (d): observed species; (e): Simpson; (f): Shannon; A: control; B: model; C: LGG; D: CSE; and E: 5‐ASA. Data represent the mean ± SD, mean values with different letters over the bars are significantly different (*p* < .05).

Alpha diversity including ACE, Chao1, PD whole‐tree, observed species, and Simpson and Shannon index was assessed by the abundance of the microbial which revealed the diversity of microbial species (Figure 3). DSS‐induced colitis, as evidenced by significantly decreased alpha index except for Simpson index, was compared to the control and treatment groups (*p* < .05). The alpha diversity of treatments was significantly different from model group. The index of LGG and CSE groups presented significant increase in the abundance of the community compared to the 5‐ASA group. Notably, the CSE group exhibited the highest value in ACE, Chao1, PD whole‐tree, observed species, and Shannon index, higher than the LGG group, which revealed significantly improved abundance and diversity of the microbial (*p* < .05).

**FIGURE 4:**
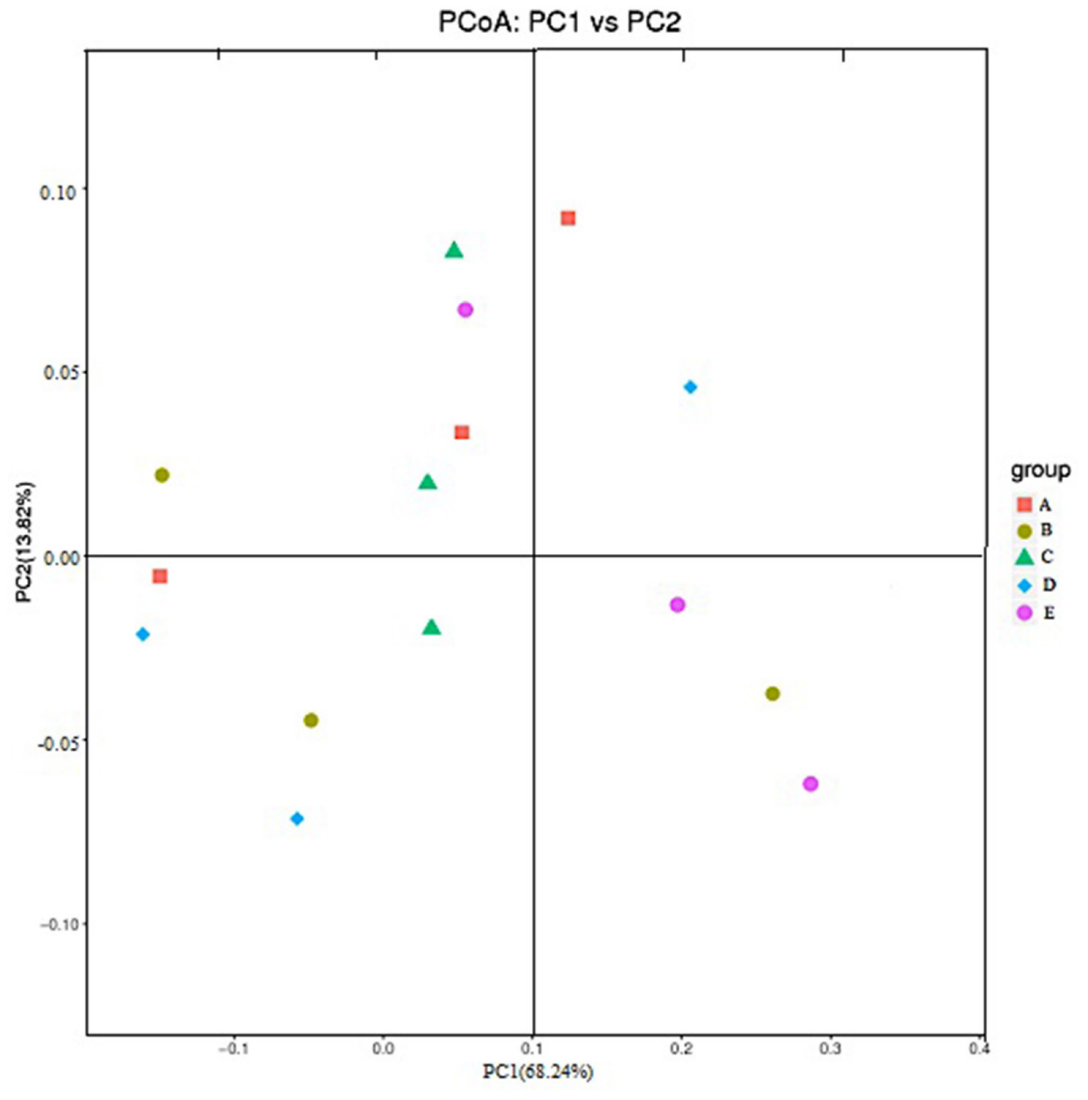
Principal component analysis of microbial communities in mice. A: control group; B: model group; C: LGG; D: CSE; and E: 5‐ASA.

The multivariable statistical method PCoA was used to analyze beta diversity (Figure 4), which evaluated the similarity and difference in microbial evolution. The weighted UniFrac matrix of diversities with different treatments at the genus level distinguished differences in species abundance. The distribution of the two principal components represented 68.24% (PC1) and 13.82% (PC2) of the accumulative variance contributions for a total of 82.06%. The microbial of the mice treated with LGG, CSE, and 5‐ASA group were separate independently, which indicated more change in structure and abundance of intestinal flora.

### 3.6 Effect of *C. salicifolius* extract on modulation of intestinal microbiota

**FIGURE 5:**
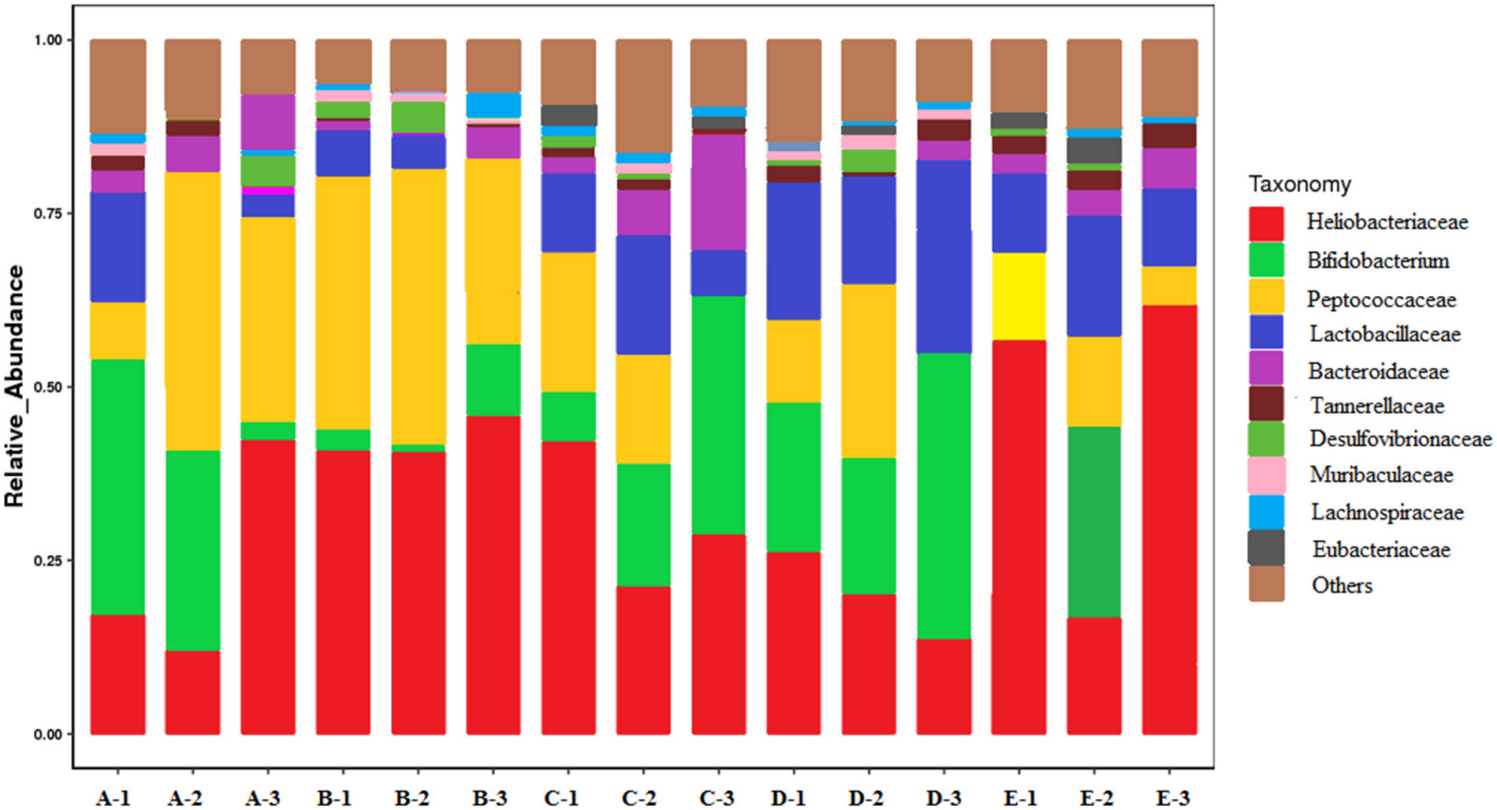
Relative abundances of the main genus in the different mice groups. A: model group; B: control group; C: LGG; D: CSE; and E: 5‐ASA.

The V3–V4 region of the 16 S rRNA gene was sequenced with Illumina Miseq to indicate the modulatory effects of different treatments on gut microbiota of DSS‐induced mice. The annotation results of species evaluated the relative abundances of each group at the genus level (Figure 5). A total of 1076 gene sequences were identified, of which 10 genera were classified to have relative abundances. The relative abundance of *Heliobacteriaceae* was greatest in the mice of the control group (23.9%), followed by several genera from *Bifidobacterium* (15.16%), *Peptococcaceae* (12.96%) *Lactobacillaceae* (11.98%), *Bacteroidaceae* (10.82%), and *Tannerellaceae* (9.65%). *Desulfovibrionaceae* and *Muribaculaceae* also accounted for large proportions (5.98% and 4.69%, respectively).

**FIGURE 6:**
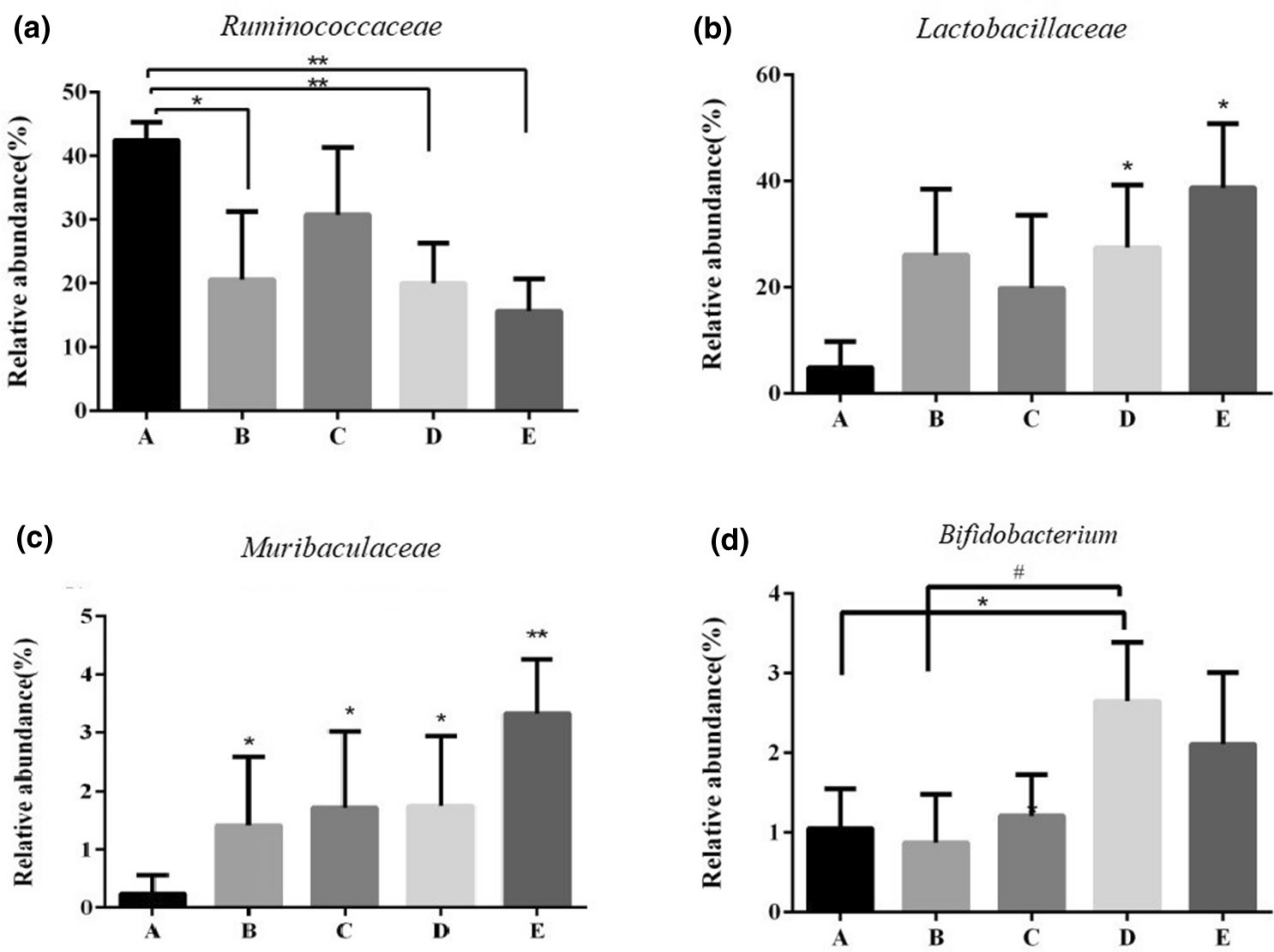
Changes in relative abundance in genus level. (a) model group; (b) control group; (c) LGG; (d) CSE; and (e) 5‐ASA.

The relative abundance showed different diversity of treatments (Figure 6). A significant decrease in the relative abundance of *Heliobacteriaceae* was observed following treatment with all treatments compared with the model group. The LGG and CSE groups exhibited extremely significant lower abundance (*p* < .05). *Heliobacteriaceae* was usually classified into *Firmicutes* and *Peptococcaceae* belonging to *Bacteroidetes*. The ratio between the two phyla was considered for certain relations with intestinal flora (Meng et al., 2020). *Lactobacillaceae* and *Bifidobacterium* were regarded as probiotic, which was beneficial for intestinal adjustment. At *Bacteroidaceae* genus level, there was no difference among LGG, 5‐ASA, and model groups, however, the abundance of CSE group was significantly reduced. After the administration of CSE and LGG groups, the relative abundances of *Bifidobacterium* increased significantly (*p* < .05); noteworthily the CES group reached the highest abundance at 39.52%. For the *Lactobacillaceae*, only the CES group markedly increased the relative abundance compared with model group and presented a maximum abundance of 1.98%.

Colitis is a common inflammatory disease that is involved with abdominal pain, diarrhea, and constipation, which seriously affects human health (Min et al., 2020). Although 5‐ASA as therapeutic drug could treat colitis, the treatment process is accompanied by side effects such as stomach distension, nausea, vomiting, and abnormal liver function. Previous studies have suggested that intestinal tract disorder and intestinal flora change was important factor for colitis. Most modern medical research on colitis has been concerned with probiotics, supplemented to relieve diarrhea, which is reported to be due to their metabolites such as bacteriocins and SCFAs (Bharucha et al., 2017). However, there have been less reports focusing on plant extract provided by natural resources in the degree of alleviation of colitis or the resulting changes in the fecal flora in mice.

*Chimonanthus salicifolius* distributed in the mountain areas, which is considered a unique plant in Eastern China, has been applied for both medicine and food. The leaves of *C. salicifolius* have been reported for drinking tea and antivirals. Functional component of this plant was mainly achieved by alcohol and aqueous extraction. In this study, main component was collected from *C. salicifolius* aqueous extraction and analyzed by HPLC instrument. They are rutin, hyperin, isoquercitrin, afzelin, and kaempferol, respectively, which is consistent with the previous study that the different chemical substances existed between aqueous and alcohol extract (Liu et al., 2013). Notably, these components belonged to flavonoids and widely existed in plants, which are capable of anti‐infection and antioxidation. Meanwhile, the alcohol extract proved to have broad inhibitory activity against most pathogenic bacteria such as *Escherichia coli* and *Staphylococcus aureus*. Ethyl acetate extract could alleviate intestinal mucositis in mice induced by 5‐fluorouracil, which may be related to the anti‐inflammatory and antioxidant effects (Wen et al., 2021). Although bacteriostatic properties of *C. salicifolius* have been reported, the research was rarely related to effect of bacteriostatic function on intestinal microecology. This study focused on intestinal tract revolution mechanism of *C. salicifolius* extract which played an important role in improving colitis.

In this study, DSS‐induced colitis model was established successfully accompanied by fecal occult blood and histological scores. The treatment groups could improve colon length and colonic epithelial structure. Remarkably, the CES group showed significantly decreased scores and increased colon length compared with LGG and 5‐ASA groups, and villi, crypts, and intestinal glandular were relatively complete. DSS upregulated the expression levels of IL‐6, IL‐8, and TNF‐α in the colon, which may lead to the immune disorder of the colon, aggravated inflammation, and damaged colon tissue. According to the inflammatory cytokines, although the LGG, 5‐ASA, and CSE group could inhibit inflammatory cytokines and increase anti‐inflammatory cytokines in colon tissues of mice, CSE group exhibited significantly increased relative expression of anti‐inflammatory cytokines IL‐10 and TGF‐β comparison to LGG and 5‐ASA groups. Moreover, the levels of SCFAs in patients with colitis were significantly reduced, suggesting a potential factor of SCFAs in the pathogenesis of colitis. Studies have shown that AA, PA, and BA could decrease the secretion of proinflammatory cytokines TNF‐α, thereby playing an anti‐inflammatory role. There was no significant difference in the level of AA, PA, IA, and VA between the LGG and CES groups; meanwhile, the CSE group produced the highest value (176.56 ± 21.78) in BA which indicated that *C. salicifolius* aqueous extract could produce SCFAs through metabolism and regulate the expression of inflammatory cytokine.

Intestinal microbiota is directly affected by gut microecological environment. It was reported that the number of beneficial bacteria such as lactic acid bacteria decreased in the colon of colitis patients and the colon environment was mainly alkaline, which promoted the expression of proinflammatory genes to induce inflammation, ultimately leading to colitis. Therefore, regulating intestinal flora and increasing the abundance and diversity of intestinal probiotics are effective methods for the treatment of colitis. In this study, the abundance of model group exhibited a high rate of *Heliobacteriaceae* and *Peptococcaceae* which belonged to the *Firmicutes* and *Bacteroidetes*, respectively. The result was the same similarity as previous research (Grigor'Eva, 2021). The treatment of LGG, 5‐ASA, and CSE groups showed a remarkable difference from the model group. The CES group dramatically increased the abundance and diversity of mice induced by DSS in both *Lactobacillaceae* and *Bifidobacterium*. Remarkably, at the genus level, the CES group presented a significant increase in *Lactobacillaceae* abundance compared to the LGG group which improved the probiotic abundance and diversity, thereby improving DSS‐induced colitis. 5‐ASA considered conventional medicine had no difference between the model groups and showed a relatively low abundance of *Lactobacillaceae*. The study revealed that extract of *C. salicifolius* had the potential to regulate intestinal microbial diversity and replace antibiotic drugs from being especially effective for relieving colitis.

## 4 CONCLUSIONS

In this study, the main components of *C. salicifolius* extract were analyzed and identified as rutin, hyperin, isoquercitrin, afzelin, and kaempferol. We investigated the effects of the administration of the extract on colitis symptoms compared with LGG and 5‐ASA. The treatment of *C. salicifolius* could significantly improve colonic mucosal injury and colon length, inhibit inflammatory cytokines, increase anti‐inflammatory levels (*p* < .05), and promote metabolic production of SCFAs. Moreover, the intervention of *C. salicifolius* extract on colitis mice effectively regulated intestinal flora induced by DSS and restored their relative abundance. Furthermore, the CES group significantly increased the abundance and diversity of *Lactobacillaceae* compared with other treatments. Therefore, the results indicate that *C. salicifolius* extract presents the potential to be developed as a candidate for treating colitis.

## FUNDING INFORMATION

This work was supported by the funds of the Major Science and Technology Projects of Zhejiang Province (No. 2020C04002); the Chinese Academy of Engineering Academy‐Locality Cooperation Project (No. 2019‐ZJ‐JS‐02); the Natural Science Foundation of Zhejiang Province (No. LR22C200005); and Zhejiang Business Collage Foundation of China (KT21001226; KT21001231).

## CONFLICT OF INTEREST STATEMENT

The authors have no conflict of interest to declare.

## Data Availability

The data that support the findings of this study are available from the corresponding author upon reasonable request.
